# Investigating Migration Inhibition and Apoptotic Effects of *Fomitopsis pinicola* Chloroform Extract on Human Colorectal Cancer SW-480 Cells

**DOI:** 10.1371/journal.pone.0101303

**Published:** 2014-07-03

**Authors:** Yaqin Wang, Xiaoxia Cheng, Pan Wang, Lu Wang, Jianping Fan, Xiaobing Wang, Quanhong Liu

**Affiliations:** 1 Key Laboratory of Medicinal Resources and Natural Pharmaceutical Chemistry, Ministry of Education, National Engineering Laboratory for Resource Developing of Endangered Chinese Crude Drugs in Northwest of China, College of Life Sciences, Shaanxi Normal University, Xi’an, Shannxi, China; 2 School of Chemistry & Chemical Engineering, Shaanxi Normal University, Xi’an, Shaanxi, China; National Cancer Center, Japan

## Abstract

**Background:**

*Fomitopsis pinicola (Sw. Ex Fr.m) Karst* (FPK) which belongs to the Basidiomycota fungal class is one of the most popular medical fungi in China. It has been used for many diseases: cancer, heart diseases, diabetes and so on. However, little study on the pro-apoptotic effect and migration inhibition of FPK chloroform extract (FPKc) has been reported and the possible involved mechanism has not been illuminated.

**Methodology/Principal Findings:**

Chemical analysis was performed by HPLC which showed ergosterol (ES) concentration was 105 µg/mg. MTT assay revealed that FPKc could selectively inhibit SW-480 cells viability with the IC_50_ of 190.28 µg/ml. Wound healing and transwell assay indicated that FPKc could inhibit the migration of SW-480 cells obviously, FPKc could also dramatically decreased the matrix metalloproteinases-2, 9 (MMP-2 and MMP-9) expression. Annexin V–FITC/PI staining, nuclear Hoechst 33342 staining and DNA fragmentation analysis revealed that FPKc and ES could induce SW-480 cells apoptosis. The apoptosis process closely involved in ROS accumulation and depletion of GSH, activation of caspase 3, poly (ADP-ribose) polymerase (PARP) degradation. FPKc could also up-regulate P53 expression and thus lead to G1 phase arrest. When SW-480 cells were pretreated with N-acetylcysteine (NAC), the ROS generation, cell viability and apoptotic ratio were partially declined, which indicated that ROS was vertical in the pro-apoptosis process induced by FPKc. Moreover, in the whole process, ES which has been previously found in FPKc had the similar effect to FPKc. Thus we could conclude that ES, as one of the highest abundant components in FPKc, might also be one of the active constituents.

**Conclusion/Significance:**

FPKc could inhibit the migration of SW-480 cells, induce SW-480 cells G1 phase arrest and cause ROS-mediated apoptosis effect. And ES might be one of the effective constituents in the whole process.

## Introduction

Colorectal cancer (CRC) is a tumor with fleetness increasing worldwide every year. Each year nearly half of the diagnosed patients would be dead from the disease [1]. CRC is considered as the third most common malignant tumor and the third cause of death by cancer in the USA [2]. Although the incidence of CRC is much lower in Asia comparing to that in the USA, it has been increasing rapidly in China [3]. While traditional treatment for CRC including surgery, radiotherapy, and current chemotherapeutic options have been out of efficiency and have many side effects [4]. All these problems highlight the importance to find out a new agent for CRC. As traditional Chinese medicine has been more and more popular, it has been regarded as potential therapeutic agent because of its high efficiency and safety [4].

*Fomitopsis pinicola (Sw. Ex Fr.) Karst* (FPK) which belongs to the Basidiomycota fungal class is one of the most common wood rooting fungi and widely distributed in many countries in the world, such as Japan, Korea, China and Sweden [5]. FPK was traditionally used as a health food source for plant growth regulation and diabetes in Japan [6], [7]. FPK as a nontoxic natural product has been more and more attractive for scholars, and its extracts have been reported to have anti-inflammatory, anti-microbial, anti-fungal and anticancer effect [8], [9], [10]. For anticancer effect of FPK, the research mainly focused on its ethyl acetate and ethanol extracts. For instance, Ren G demonstrated both petrol ether and ethyl acetate extracts of FPK have the cytotoxicity against some tumor cell lines such as Hela and SMMC-7721 [11]. Hung-Tsung Wu from Taiwan has demonstrated F. pinicola ethanol extract has anticancer effect on S180 cells in vitro and in vivo. He also proves that it could trigger Homo sapiens hepatoma (HepG2), lung cancer (A549), colorectal cancer (HCT-116) and breast cancer (MDA-MB-231) cells apoptosis [12]. And for FPK chloroform extract (FPKc), there is only one report to demonstrate its anti-fungal effect [10]. To our best knowledge, little information about the anticancer effect of FPKc has been published. Therefore, the first aim of our study was to evaluate whether FPKc can exert its anticancer effect in our experimental system, then mainly focus on investigating the migration inhibition and pro-apoptosis effect of FPKc and the potential involved mechanisms.

**Figure 1:**
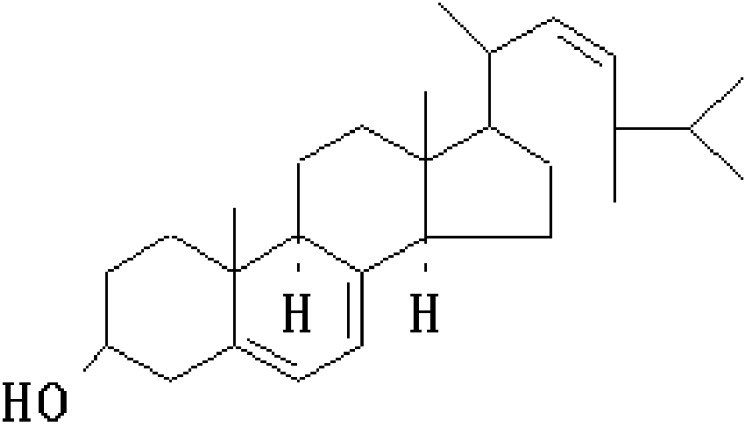
Chemical structure of ergosterol.

Further, the chemical analysis of FPK extracts, which mainly point the n-hexane and methanol extracts of FPK contain some triterpenoids such as ergosterol, ergosterol derivatives, lanostane triterpenes and so on [13], [14]. While the chemical analysis about FPKc has never been studied. Because ergosterol (ES, Figure 1) has been reported to widely distribute in many kinds of fungi and show some anticancer effect [15], [16]. Thus the other aim of this study was to explore the chemical components of FPKc and investigate whether ES worked when FPKc carried out its anticancer effect.

## Methods and Materials

### Collection and preparation of chloroform extract

No specific permissions were required for the location where FPK was collected and this study did not involve endangered or protected species.

The fresh FPK was collected in July 2011 from Pingheliang, the south of QinLing Mountains, Shaanxi province, China (latitude, 33°27′N; longitude, 108°30′E; altitude, 2305 m). It was authenticated by Prof. Yaping Xiao and deposited in the Ministry of Education, Key Laboratory for Medicinal Plant Resource (MPR) and Natural Pharmaceutical Chemistry, Shaanxi Normal University, Xi’an, Shaanxi, P.R. China.

The ethanol extract of FPK was obtained through the ultrasonic extraction method and then concentrated with a rotary evaporator (RE-2000 A; Belong, Shanghai, China). Thirdly, it was dried with a freeze-dryer (ALPHA1–2, CHRIST, Germany) and finally lyophilized. The ethanol extract was then fractionated by chloroform (CHCl_3_). The chloroform fraction was homogenized in 70% ethanol and the supernatant was filtered using 0.22 mm filters.

### HPLC analysis

The determination of FPKc and ES was evaluated through the high performance liquid chromatography (HPLC) analytical method. The LC system consisted of Shimadzu LC-20AT (Shimadzu Corp., Kyoto, Japan) with a quaternary pump, a thermostat column compartment and Shimadzu LC solution software. Separation of phytochemicals was achieved on a Shim-pack VP-ODS C18 column (Shimadzu, 1504.6 mm; 5 mm particle size). The mobile phase consisted of acetonitrile and water. A gradient elution program was used as 10–100% acetonitrile (v/v) at 0–80 min, 100%–85% at 80–90 min, keeping 85% at 90–100 min. The column temperature was kept constantly at 40°C, and the mobile phase flow rate was 0.8 ml/min. The detection wavelength was 254 nm and 20 µl of samples were injected. Re-equilibration duration was 15 min between individual runs.

### Calibration curves

ES standard was brought in Sima, Tianjin, China. The purity was shown to be greater than 98%. Calibration curves were constructed with dilutions of 2000, 1000, 500, 250, 125 µg/ml in methanol. A volume of 20 µl was injected by triplicate and calibration curves were based on the average peak areas of each chromatogram. The calibration curves showed an R^2^ of 0.993 for ES.

### Cell culture

The SW-480, SW-620, Caco-2 and HEK-293 cells were purchased from the cell bank of the Chinese Academy of Science, Shanghai, China. The SW-480, SW-620 Caco-2 and HEK-293 cell lines were cultured in RPMI-1640, L-15 and DMEM medium, respectively. All of them were cultured with 10% fetal bovine serum (FBS), 1% penicillin–streptomycin (100 U/ml penicillin and 100 µg/ml streptomycin) and 1% glutamine in 100 cm^2^ tissue culture flasks under a humidified 5% CO_2_ and 95% air atmosphere at 37°C.

### Cell viability

To evaluate the effect of FPKc on SW-480, SW-620 and Caco-2 cell viability, cells were seeded in 96-well plates (5×10^4,^ 1×10^5^ and 1×10^5^). Various concentrations of FPKc were used on SW-480 (120, 160, 200, 240 µg/ml, 70% ethanol was used as the solvent control) and SW-620 (40, 80, 120, 160, 200, 240 µg/ml) and Caco-2 (40, 80, 120, 160, 200, 240, 280 µg/ml) cells. Different doses of ES (0, 12, 24 µg/ml; 100% ethanol) were added into SW-480 cells. After that all the cells were incubated for 48 and 72 h, respectively. Human Embryonic Kidney *293* (HEK-293) cells were used as normal cells by contrast to evaluate the cytotoxic anticancer activity of FPKc. The viability of the four cell lines was determined by using MTT assay [17]. The absorbance at 570 nm was recorded using a microplate reader (Bio-Tek ELX800, USA). The cell viability of FPKc and ES treated samples was then obtained by comparing to the control. (All the concentration mentioned in this article referred to the dry weight).

### Cell motility

Cell motility was evaluated by scratch wound and transwell assay. For the scratch wound assay: SW-480 cells were plated in 24-well plates for 24 h, then cells in individual wells were wounded by scratching with a pipette tip and the cells were incubated with the indicated concentration of FPKc and ES for 12 and 24 h. The cells were photographed under phase-contrast microscopy (×200 magnification).

For the transwell assay, 5×10^5^ cells were seeded in top chamber with serum-free medium containing 0.3% BSA and medium containing 10% serum was added to the lower chamber of the Corning chamber (polycarbonate filter with 8-mm pore size inserts, Corning Pharmingen, San Diego, CA). After incubation for 36 h, cells moved to the underside of the membrane were detected by wiping the upper side with cotton swab and staining the underside cells with 0.1% crystal violet solution. Cells moved to the underside of the membrane were observed by microscope, and the crystal violet adhered in the underside cells were dissolved in 33% acetic acid, the OD ratio of the solution was measured at 570 nm by microplate reader.

### Immunofluorescence

After FPKc incubation for 24 h, cells were disposed as folowing: fixed with 4% paraformaldehyde, permeabilized with 0.1% Triton X-100 and blocked with 5% bovine serum albumin (BSA), between each step cells were washed by PBS for three times. After cells were blocked, they were incubated with anti-MMP-9 and -MMP-2 antibodies (purchased from Santa Cruz) overnight and dyed with the corresponding secondary antibody performed by immunoglobulin FITC (Zhong Shan Golden Bridge Biotechnology Co., Beijing, China) at 37°C in the dark for 1 h, and then Cells were imaged with fluorescence microscope (Nikon E 600).

### Hoechst 33342 staining

Hoechst 33342 staining was performed to detect alterations of nuclei morphology of SW-480 cells after FPKc and ES treatment. The treated cells were stained by 10 µM Hoechst 33342 for 15 min at 37°C, then the stained cells were washed three times with PBS and observed using a fluorescence microscopy with standard excitation filters (Nikon, Japan). Excitation wavelength was 346 nm and emission wavelength was 460 nm.

### Flow cytometry analysis of DNA fragmentation

The method to analyze DNA fragmentation was flow fluorocytometric detection of DNA hypoploidy after adding propidium iodide (PI; Sigma, St. Louis, USA) to the dying cells and permeabilizing them by freeze-thawing [18]. To investigate the effect of FPKc and ES on DNA damage of SW-480 and HEK-293 cells, we performed oligonucleosomal DNA fragmentation by flow fluorocytometry. Cells in 24-well plates were treated with various concentrations of FPKc and ES for 12 h, respectively. Cells were then stained with 5 µg/ml PI and analyzed for DNA content by using flow cytometry.

### Cell cycle analysis

SW-480 were seeded in 24-well plates, and then treated with FPKc and ES (0, 240, and 24 µg/ml) for 24 h. Then cells were harvested and disposed as following steps: washed twice with cold PBS containing 1% BSA (Sigma, St. Louis, USA), fixed with 70% ice-cold ethanol at −20°C overnight, then washed twice with cold PBS, incubated with 100 µg/ml RNase A (Sigma, St. Louis, USA) for 30 min at 37°C, after that stained with 50 µg/ml PI for 30 min in the dark and finally analyzed by flow cytometry (Millipore, USA).

### Annexin V–FITC/PI staining experiment

Phosphatidylserine serves as a sensitive marker of cells undergoing apoptosis when it is externalized to the outer leaflet [19]. Thus the ratio of apoptotic cells was measured with an Annexin V–FITC Apoptosis Detection Kit (Invitrogen, USA) according to the manufacturer’s protocol. Briefly, SW-480, SW-620 and HEK-293 cells were treated with various concentrations of FPKc and ES for 24 h at 37°C, then the treated cells were harvested and re-suspended in 200 µl binding buffer. After adding 2 µl Annexin V–FITC and 2 µl PI into the cell suspension, the samples were incubated for 15 min at room temperature in the dark. The apoptotic index was immediately determined by flow cytometry.

### Detection of intracellular reactive oxygen species (ROS) generation

Some edible fungi, such as Pleurotus abalonus, could provoke ROS-mediated apoptosis [20]. In this study we also measured changes of the cellular ROS level through the oxidative conversion of the sensitive fluorescent probe 2′, 7′-dichlorofluorescein-diacetate (DCFH-DA) to fluorescent 2′, 7′-dichlorofluorescein (DCF). DCFH-DA readily diffuses through the cell membrane and is enzymatically hydrolyzed by intracellular esterases to form non-fluorescent DCFH, which is then rapidly oxidized to form highly fluorescent DCF in the presence of ROS, and the fluorescence intensity is proportional to ROS production. **SW-480** and HEK-293 cells in 24-well plates were treated with the mentioned concentration of FPKc and ES for 3 and 6 h (HEK-293 cells only for 6 h). The cells were harvested and washed twice with PBS, re-suspended in 500 µl of 10 µM DCFH-DA (purchased from Molecular Probes Inc., Invitrogen, CA, USA**)** and incubated at 37°C for 30 min in the dark. The samples were then immediately detected by flow cytometry. Histograms were analyzed using FCS Express V3.

### Glutathione determination

SW-480 cells were incubated in 24-well plates, and then they were treated with FPKc and ES for 3 h and 5 h. After that, the treated cells were harvested and washed twice with PBS. Total glutathione concentrations were conducted by Glutathione Kit (Nanjing jiancheng bioengineering institute, China) according to the manufacture’s protocol. At last, the samples were measured at 405 nm with microplate reader (Bio-Tek ELX 800, USA).

### Western blotting staining

SDS–PAGE and immunoblotting were performed according to standard procedures. Briefly, after treated with FPKc (240 µg/ml) and ES (24 µg/ml) for 0 h, 12 h, 24 h and 48 h, cells were lysed by RIPA buffer on ice. The protein samples were separated on a 10% SDS polyacrylamide gel, and then the gel was transferred to nitrocellulose membranes (Millipore, MA, USA) and blotted with primary antibodies (Caspase-3, Cleaved-RARP, Bcl-2, P53 were purchased from Cell Signaling Technology, USA) overnight at 4°C. The bound primary antibodies were then tagged with IRDye 680 Conjugated IgG (Li-cor, Biosciences) at room temperature for 1 h. And the infrared fluorescence was detected with the Odyssey infrared imaging system (Li-Cor Bioscience, Lincoln, NE).

### Statistical analysis

All the experiments were performed in triplicate, and data were expressed as means ± SD. IC50 values were calculated by regression analysis. The data were subjected to an analysis of Duncan’s multiple range test (SPSS, version 18.0). A significant difference was judged to exist at a level of **p<0.01.

## Results

### HPLC

**Figure 2:**
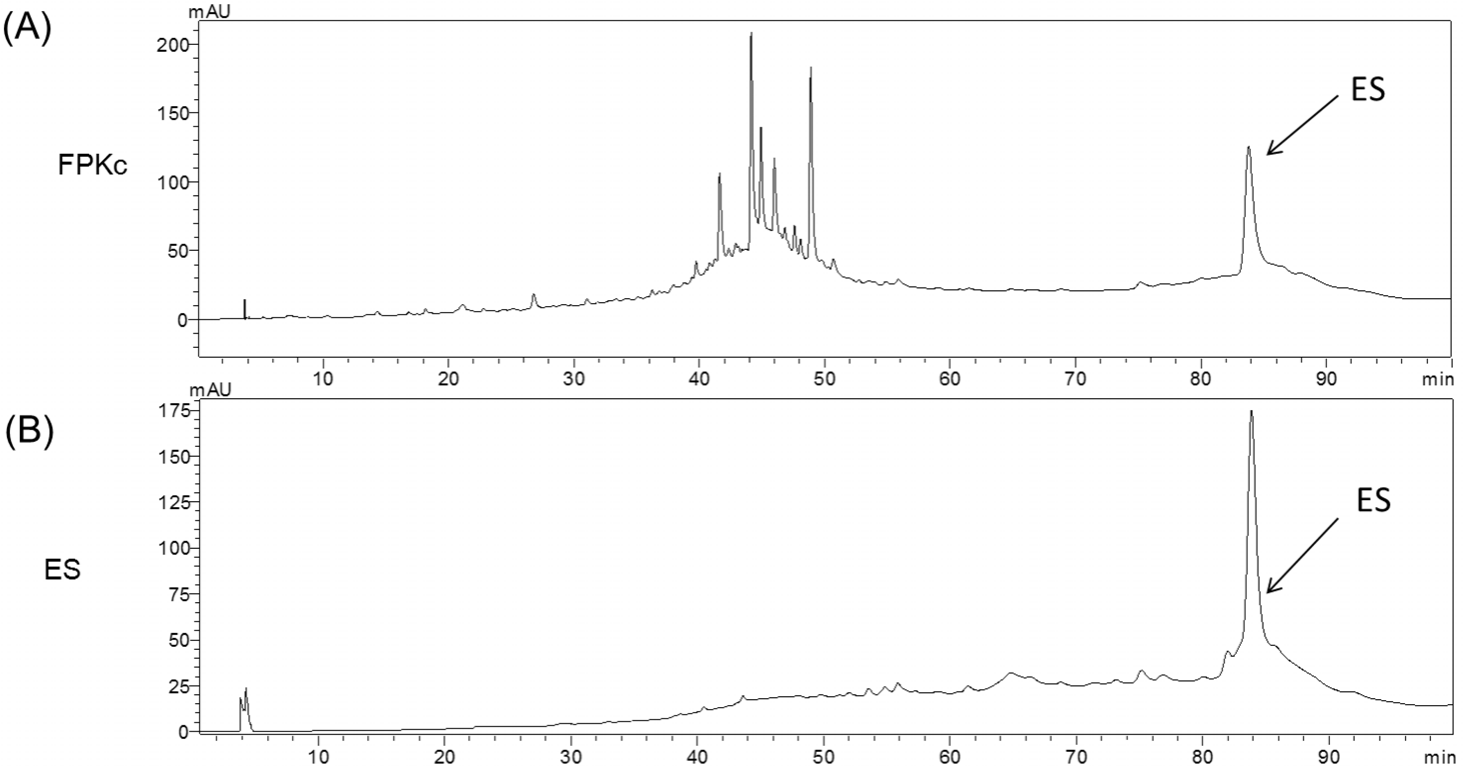
The HPLC chromatograms of FPKc (A), standard ergosterol (B). FPKc and ES standard were identified by HPLC-PDA at 254 nm as described in the experimental section.

HPLC assay has been accessed to identify ES and the chemical components of FPKc. The data were shown in Figure 2, at the same experimental conditions, ES standard showed its retention time at 83.8 min (Figure 2B); FPKc displayed six main peaks and included a peak with the same retention time of ES, implying ES might be one of the main components of FPKc (Figure 2A); From the area of the peaks, it revealed ES ranked the second in FPKc; From the quantitative determination of ES in FPKc with HPLC, we speculated ES possessed 105 µg/mg (about 10% in the total FPKc).

### Cytotoxic effects of FPKc and ES

**Figure 3:**
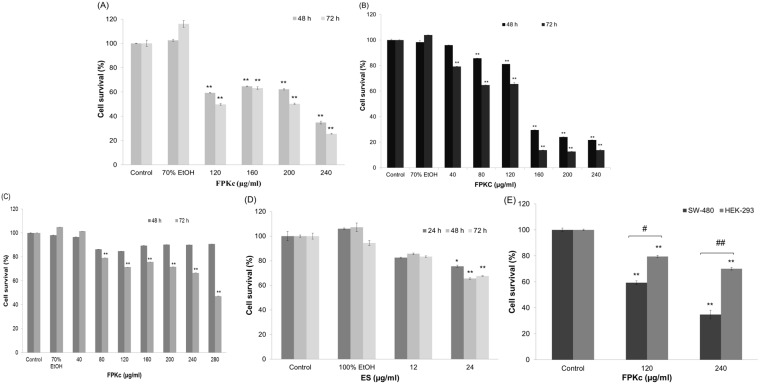
Cell cytotoxicity. SW-480, SW-620, Caco-2 and HEK-293 cells viability after FPKc (A, B, C, D) and ES (E) treatment was measured by MTT assay. Each value was expressed as a mean ± S. D. of at least three independent determinations. One-way ANOVA was used for comparisons of multiple group means followed by Dunnett’s t-test. *P<0.05 and **P<0.01 versus the control. (error bars = S. D., n = 3).

Figure 3A–C showed the cytotoxicity of FPKc on SW-480, SW-620 and Caco-2 cells respectively which was in a dose- and time- dependent manner. When SW-480 cells were treated with 120 and 240 µg/ml FPKc for 48 h, the cell viability loss was 34.99±1.08% and 65.20±2.34%, the IC_50_ value was calculated as 190.28 µg/ml; For SW-620 cells, the cell viability declined to 74.61±0.99% and 29.52±1.28% when the concentration was 80 and 160 µg/ml, respectively, the IC_50_ value was calculated as 143.26 µg/ml. Caco-2 performed less sensitive than the above 2 cell lines. After 72 h incubation with FPKc, Caco-2 started to perform viability loss, the cell viability was 71.65±0.003% with 200 µg/ml FPKc, and when the dose increased to 280 µg/ml the cell viability decreased to 47.16±0.011%, and the IC_50_ was 371.5 µg/ml.

Figure 3D showed the cytotoxic activity of ES, and cells damage was 34.52±0.58% when ES dose was 24 µg/ml after 48 h incubation. By comparison, under the same experimental conditions, 240 µg/ml FPKc caused 65.20±2.34% cell viability loss, suggesting some other cytotoxic components existing in FPKc.

For comparison, Figure 3E reflected the cytotoxicity of FPKc on human normal Embryonic Kidney 293 cells (HEK-293), a relatively weaker cell damage was observed in HEK-293 cells compared with SW-480 cells under the same dose of FPKc, suggesting FPKc has some selective tumor cell killing effect.

### Migration inhibition of FPKc and ES on SW-480 cells in vitro

**Figure 4:**
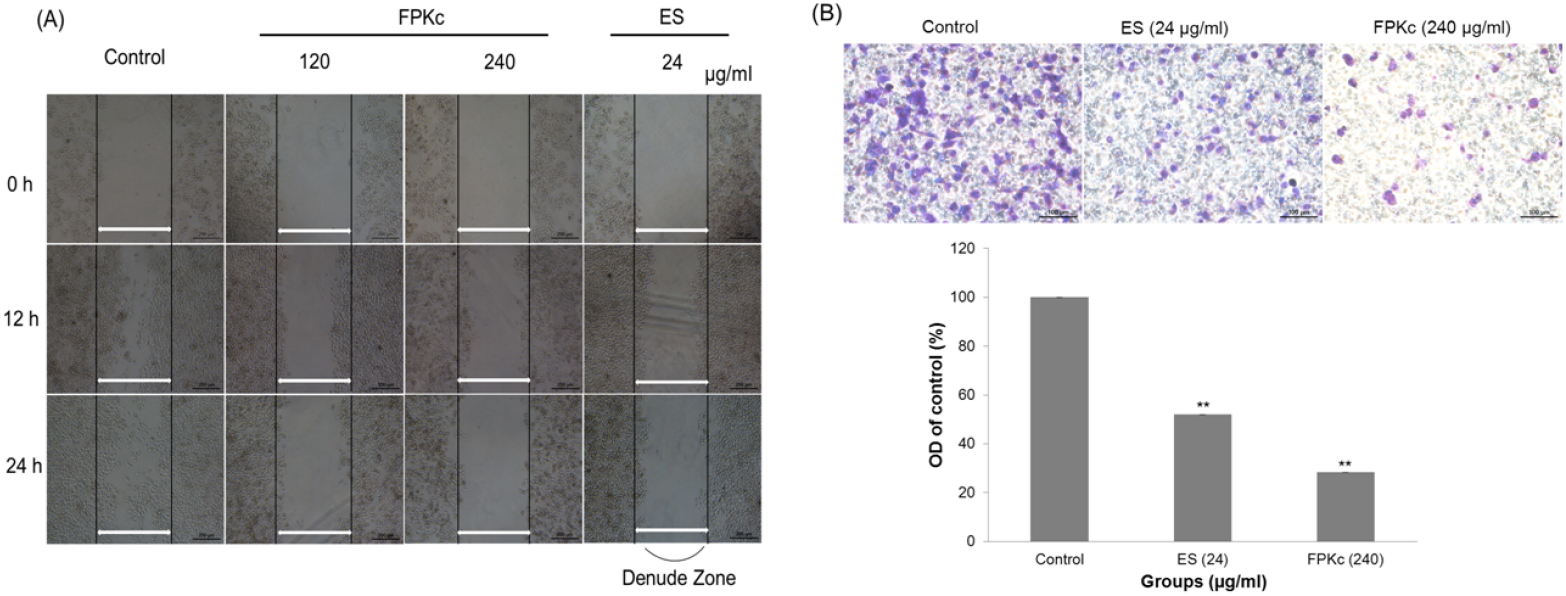
Effects of FPKc and ES on the migration of SW-480 cells in vitro. Figure 4A, Detection of cell migration ability after different treatments using wound healing assay. SW-480 cells in 24-well plates were wounded by scratching with a pipette tip and the cells were incubated with FPKc and ES for 12, 24 hours. The cells were photographed under phase-contrast microscopy (×200 magnification). **Figure 4B** **, Analysis of change in migration on SW-480 cells by transwell assay.** Cells in each group move to the lower surface of the filter were stained with crystal violet and photographed under a light microscope at ×200. b) The OD ratio of crystal violet was measured. Error bars represent SD of the means from three independent experiments. *p<0.05 and **p<0.01 versus untreated control.

To determine whether FPKc affected the migration ability of SW-480 cells, wound healing and transwell assay were conducted (Figure 4A). The wound healing ability of cells reflected their movement and migration on the surface on which they were anchored to for growth. In SW-480 cells, compared with 0 h after wounding, after 12 h of incubation, every dense cells in control gradually grew to the interspace of wound; cells in 120 µg/ml FPKc treated group showed slight difference with control; while cells in 240 µg/ml FPKc and 24 µg/ml ES treated groups rarely grew to the interspace of wound. When the incubation time increased to 24 h, the ability of cells migration was decreased with each dose of FPKc. And the number of cells with 120 µg/ml FPKc and 24 µg/ml ES did not change much comparing to the control, while the 240 µg/ml treated group decreased visibly.

Transwell assay was employed to further confirm migration inhibition induced by FPKc on SW-480 cells. And Figure 4B indicated that after 24 h incubation with FPKc, the cell migration ability decreased to 28.28±0.07% comparing to the control; and for the ES group, the migration was 51.92±0.85%, which confirmed the wound healing assay. The both results indicated FPKc and ES could inhibit the cell migration obviously.

### Immunofluorescence

**Figure 5:**
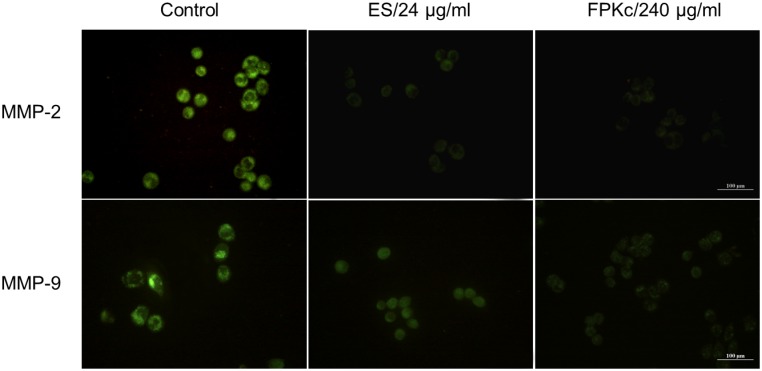
Measurement of MMP-2 and MMP-9 expression level in SW-480 cells after FPKc treatment. SW-480 cells were fixed and processed for immunofluorescence, MMP-9 and MMP-2 were visualized using FITC-label second antibody (green). Scale bars, 100 µm.

MMPs are vertical in the cell migration and movement. MMP-2 and MMP-9 were detected by immunofluorescence experiment in this study. Figure 5 revealed MMP-2 and MMP-9 were high expressed with bright green fluorescence in control group. And for the ES and FPKc groups, both enzymes decreased sharply compared to the control.

### Morphological changes induced by FPKc and ES on SW-480 cells

**Figure 6:**
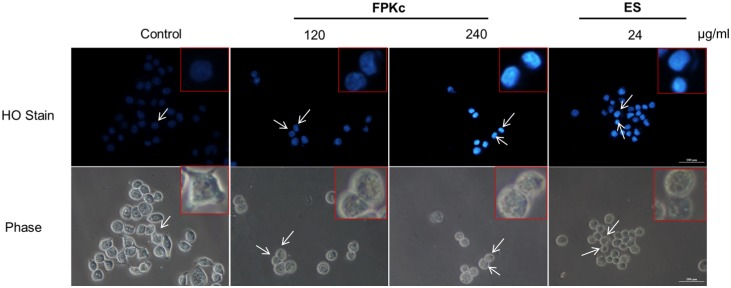
FPKc and ES effects on the cell morphology and nucleus in SW-480 cells. SW-480 cells treated for 48 h were stained with Hoechst 33342. Morphological changes were observed under fluorescent microscope.

Morphological examination was performed by Hoechst 33342. As shown in Figure 6, the nuclei of control cells were uniformly stained, and the contrast phase indicated normal SW-480 cell morphology with small islands of epithelial cells. However cells after FPKc and ES treatment for 48 h showed significant morphological changes: condensed chromatin and fragmented punctuate blue nuclear fluorescence were seen in a dose-dependent manner. When the FPKc dose was 240 µg/ml, the nuclear staining was obviously and the phase images revealed that cells changed into abnormal round type, and the number of cells was reduced distinctly.

### The DNA fragmentation induced by FPKc and ES

**Figure 7:**
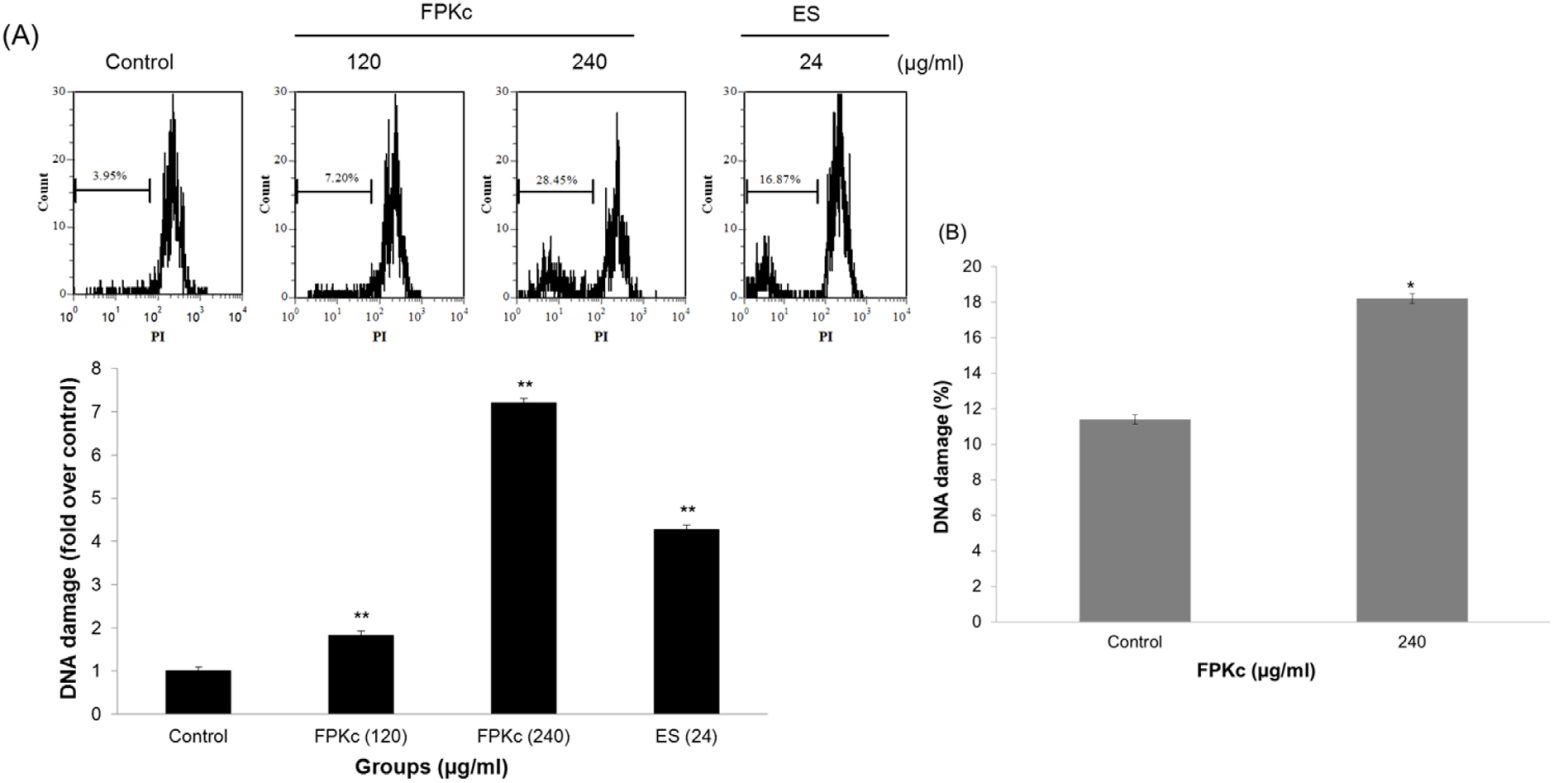
Effects of FPKc and ES on DNA fragmentation of SW-480 (A) and HEK-293 (B) cells. Both Cells were treated with FPKc and ES for 12(PI) and analyzed by flow cytometry.

PI staining by flow cytometry was used to evaluate the DNA damage caused by FPKc and ES. As displayed in Figure 7A, FPKc at 120 µg/ml triggered an 1.8-fold increase in DNA damage in SW-480 cells, and 240 µg/ml of FPKc led to a concentration-dependent increase of DNA fragmentation by 7.2-fold, compared to untreated cells (p<0.01). A similar increase by 4.2-fold in red fluorescence intensity of SW-480 cells was also obtained via the incubation with ES (24 µg/ml). Figure 7B showed 240 µg/ml FPKc induced 18.26±0.28% DNA damage on HEK-293 (about 1.6 fold of control) which indicated HEK-293 performed much less DNA damage (p>0.01) than that of SW-480 cells (p<<0.01) at the same dose of FPKc treatment.

### Cell cycle arrest induced by FPKc and ES

**Figure 8:**
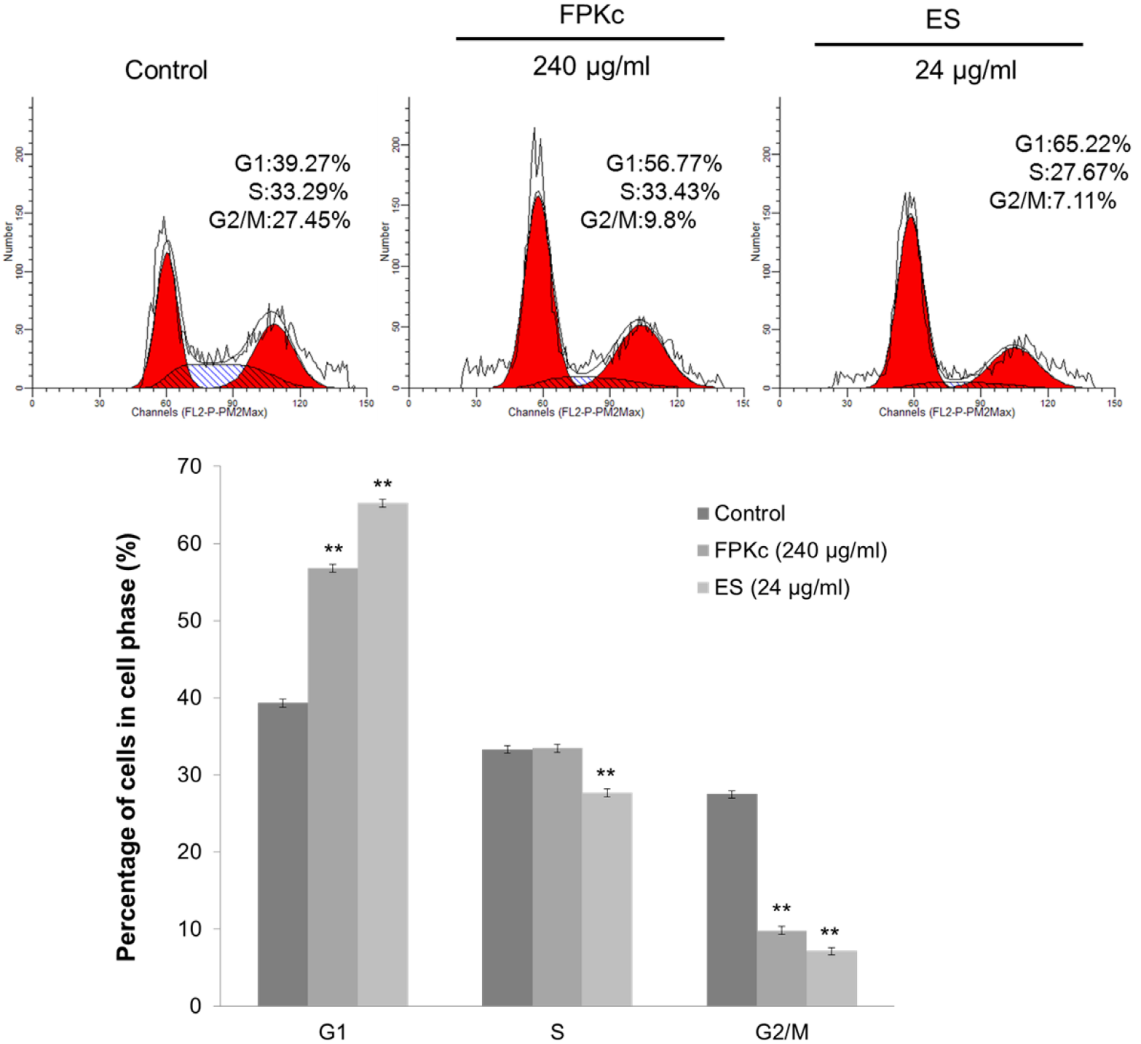
Cell cycle analysis of FPKc and ES-treated cells. SW-480 cells were harvested and fixed in 70% alcohol and then stained with PI. Finally the stained cells were analyzed using a flow cytometer.

For treating cancer, cell cycle arrest has been regarded as one of the most important targets. As we all know, cancer cells always keep unrestrained cell proliferation because their gene mutation which controlled cell division [21]. To evaluate the effect of FPKc treatment on the distribution of cells in the cell cycle, we conducted DNA cell cycle analysis by flow cytometry. Figure 8 showed the effects of FPKc and ES on the cell cycle phase (G1, S, and G2/M) distribution of SW-480 cells. After FPKc treating 24 h, the accumulation of SW-480 cells in the G1 increased from 39.27±0.56% to 56.77±0.5%; while to the ES treatment, the accumulation was up to 65.22±0.54%. The results showed that FPKc and ES could induce SW-480 cells cell cycle arrest in the G1 phase.

### Apoptosis effect induced by FPKc and ES

**Figure 9:**
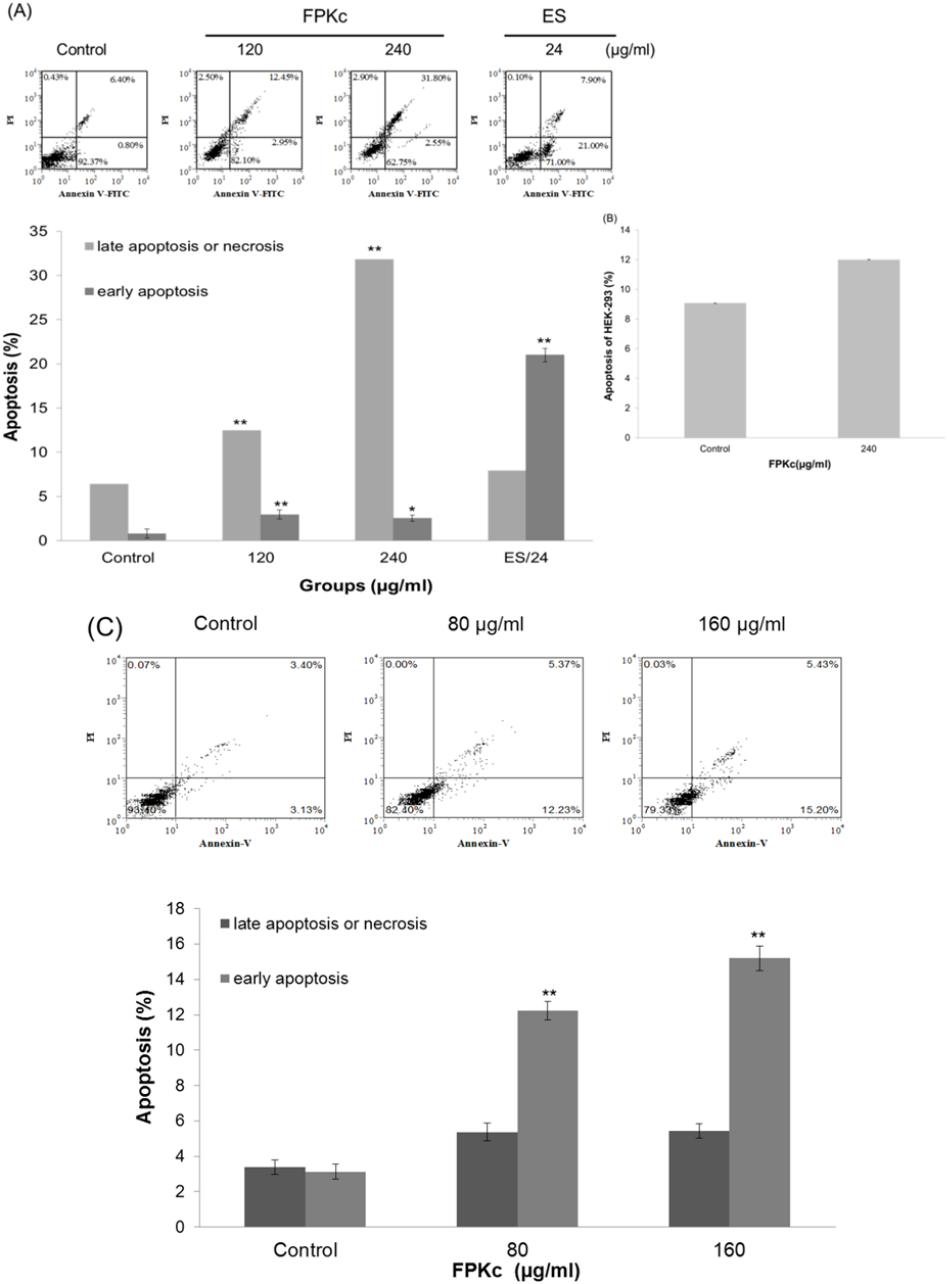
FPKc and ES induced apoptosis on SW-480 (A), HEK-293 (B), and SW-620 cells (C). Cells were double-stained with Annexin V-FITC and PI, and then analyzed by flow cytometry. All experiments were done independently in triplicate per experimental point, and representative results were shown. The results represented the mean±SD of three independent experiments. *p<0.05 and **p<0.01 indicated statistically significant differences versus control group.

Cell cycle arrest is closely related to apoptosis, and disruption of cell cycle progression may eventually lead to apoptotic/necrotic death [22]. To further evaluate the apoptosis index that FPKc and ES could provoke, the Annexin V–FITC/PI double staining was used. From Figure 9A, it was clear to see FPKc could trigger SW-480 cells apoptosis in a dose-dependent manner after incubating for 24 h. The late apoptosis ratio (upper right) increased from 15.40±0.53% to 31.82±0.93% accompanied by the increase of FPKc concentration from 120 to 240 µg/ml, while the control was only 6.42±0.5%. Interestingly, ES (24 µg/ml) could also induce phosphatidylserine externalization, the ratio of late and early phage apoptosis was 28.90±0.63% (upper and lower right).

Figure 9B showed the apoptosis led by FPKc on HEK-293 cells. After incubation with 240 µg/ml FPKc for 24 h, apoptosis rate of the treated cells was 11.83±3.2% and control group was 9.63±3.7%, which revealed there was no much difference on the two groups.

Herein SW-620 cells were also tested by AnnexinV/PI assay, and Figure 9C revealed that FPKc could induce SW-620 cells apoptosis especially early apoptosis. After 24 h incubation with FPKc, the ratio of early apoptosis cells were from 3.13±0.40% to 12.23±0.51% and 15.20±0.40% as the FPKc dose increased from 0 to 80 and 160 µg/ml.

### ROS accumulation induced by FPKc and ES on SW480 cells

**Figure 10:**
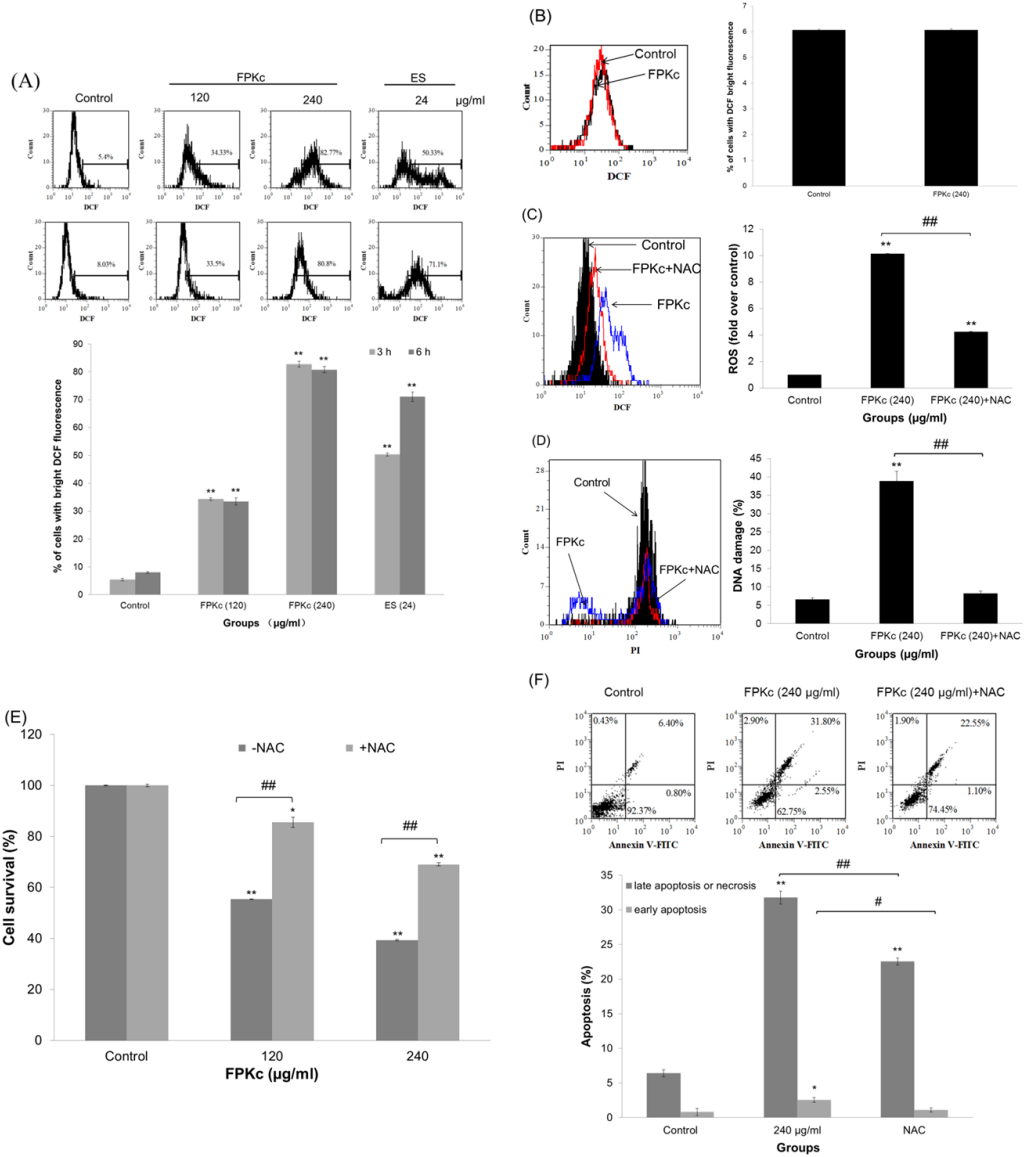
ROS generation triggered by FPKc and ES. SW-480 (A) and HEK-293 (B) cells were treated with FPKc and ES, and the ROS levels were measured by flow cytometry after staining with DCFH-DA. SW-480 cells were pretreated with NAC (5 mM) for 1 h, then intracellular ROS generation (C), DNA damage (D), cell viability (E) and apoptosis (F) were detected.

The intracellular ROS production was analyzed by flow cytometry with DCF staining. The data shown in Figure 10A suggested the intracellular ROS levels were increased after FPKc and ES treatment. At 3 h, about 34.33±0.45%, 82.77±1.05% and 50.33±0.53% of cells in 120 and 240 µg/ml FPKc and 24 µg/ml ES treated groups showed bright DCF fluorescence, while only 5.40±0.45% of cells in control group showed bright DCF fluorescence. When the incubation time increased to 6 h, the percentage of cells with bright DCF fluorescence did not change much in FPKc treated cells, ES treated cells increased to 71.10±1.7%. And Figure 10B showed after FPKc treatment, HEK-293 showed little ROS accumulation comparing to the control.

To further validate that ROS was involved in FPKc induced apoptotic effect of SW-480 cells, ROS scavengers-NAC was pretreated with SW-480 cells. As expected, in the presence of 5 mM antioxidant NAC, the accumulation of ROS decreased to 4.26 fold over the control, while FPKc group was 10.15 fold over the control (Figure 10C).

It has been reported that excessive amounts of ROS can cause oxidative damage to lipids, proteins and DNA, leading to tumorigenesis or cell death [23]. In this study, we measured DNA damage after co-treatment with NAC. And the results showed that DNA damage could be obviously reversed by NAC: DNA damage index was 38.85±2.7% when cells was treated with 240 µg/ml FPKc for 24 h, the NAC co-treatment group was only 8.20±0.71%, while the control was only 6.50±0.5% (Figure 10D). The results revealed that FPKc-induced DNA damage might be associated with ROS accumulation.

The cytotoxicity effect of FPKc on SW-480 cells was largely reversed by NAC (p<0.01, Figure 10E). The viable cells was about 85.73±0.14% and 69.62±0.21% by pretreatment with NAC, compared with about 55.42±2.00% and 39.44±0.64% by treatment with 120 and 240 µg/ml FPKc, respectively.

Annexin V-FITC/PI double staining assay also revealed that the pretreatment with NAC could partially protect SW-480 cells from FPKc induced apoptosis (Figure 10F). These results indicated that the accumulation of intracellular ROS participated in FPKc-induced apoptosis of SW480 cells.

### Alterations of intracellular glutathione concentration caused by FPKc

**Figure 11:**
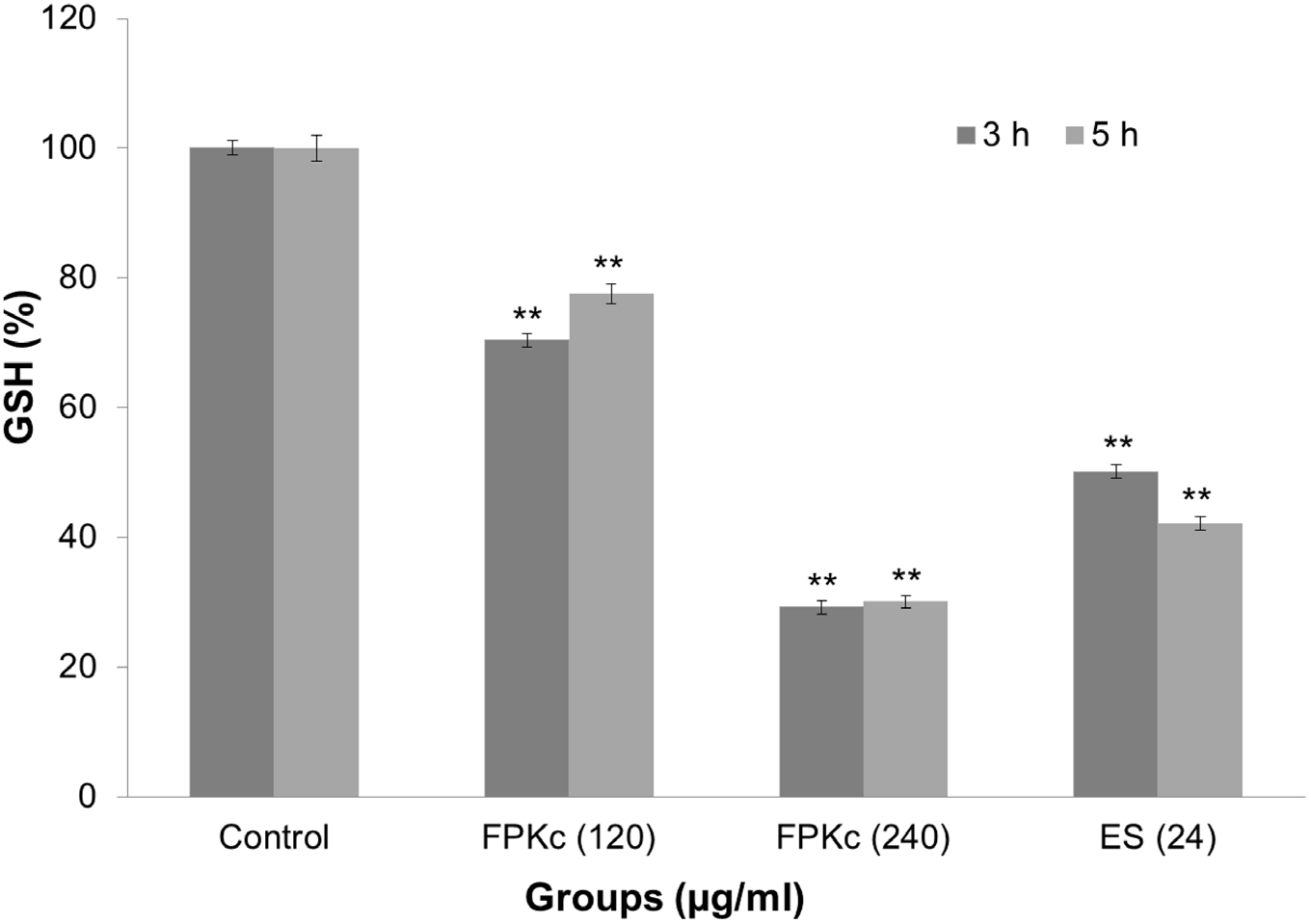
Alterations of cellular GSH levels after treatment with FPKc and ES. Intracellular GSH concentration of SW-480 cells after FPKc and ES treatments was measured at 405 nm with microplate reader.

As GSH depletion has been regarded as one of the important factor causing the accumulation of reactive oxygen species (ROS) [24], the concentration of GSH in SW-480 cells was evaluated after FPKc and ES treatment (Figure 11). When the cells were treated for 3 h, the intracellular GSH concentration decreased to 70.38±1.50%, 29.23±1.00% and 50.14±1.70% of control with 120, 240 µg/ml FPKc and 24 µg/ml ES, respectively. And when the incubation time increased to 5 h, the GSH content in SW-480 cells did not change much after FPKc treatment; while for the ES treated samples, cellular GSH decreased to 42.18±1.00%, which was in accordance with ROS generation.

### Examination of the levels of proteins associated with cell cycle and apoptosis

**Figure 12:**
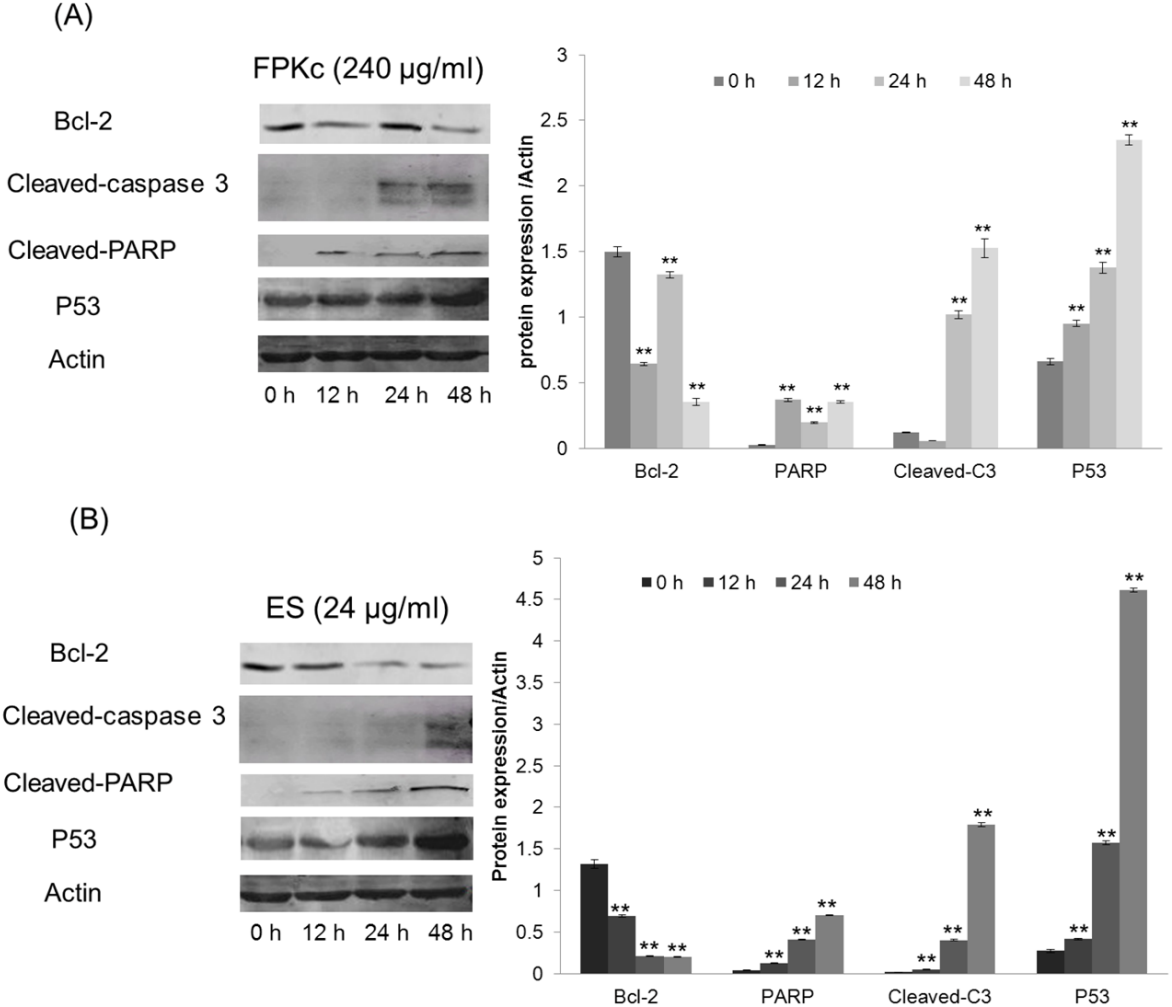
Effects of FPKc (A) and ES (B) on the expression of proteins associated with cell cycle and apoptosis in SW-480 cells. SW-480 cells were treated with 240 µg/ml FPKc and 24 µg/ml ES for 12, 24, 48 h. Western blot analysis was performed in triplicate per experimental point; Actin was used as reference control.

The underlying mechanism of FPKc-induced alteration of the protein expression involved in the cell cycle and apoptosis in the SW-480 cells was further elucidated by Western blotting assay (Figure 12). The levels of Actin served as an internal control. It was found that the expression of the anti-apoptotic protein Bcl-2 was decreased when the cells were treated with 240 µg/ml FPKc for 48 h; and to the ES (24 µg/ml) treating cells, Bcl-2 level was decreased when incubated for 24 and 48 h. In this study, cleaved caspase-3 and cleaved PARP were evaluated, and the results showed both of them were upregulated after incubated with FPKc and ES for 24 h and 48 h. Here, we also found P53 protein level increased in time-dependent manner after FPKc and ES treatment.

## Discussion

FPK as one of the most popular medical fungi in China has been widely used for many diseases including cancer in folk. According to our previous study, we had found the antitumor effect of FPKc was more efficiency than that of other fractions (data not shown). Here we choose FPKc to illuminate its anticancer activity and its possible mechanisms on SW-480 cells.

It has been well documented that n-hexane and methanol extracts of FPK contain ergisterol and ergosterol derivates [13]. While for FPKc, there was little study on its chemical analysis. Thus, in our study, we used HPLC assay to analyze the constituents in FPKc. And we have found there were 6 main peaks in it. We also chose ES as a standard to calibrate FPKc and the results implied ES might be one of main constituents in FPKc and occupied about 10.5%. Meanwhile, ES has been reported to have the anticancer effect. Thus we tested FPKc and ES to demonstrate if ES worked when FPKc exerted its anticancer effect.

In this study, we chose three kinds of human colon cancer cells SW-480, SW-620 and Caco-2 to demonstrate its general cytotoxicity. The cytotoxicity experiment revealed FPKc could distinctly reduce the number of SW-480, SW-620 and Caco-2 cells, and Caco-2 performed less sensitive than the other two cell lines. It has been reported that human colon cell lines SW-480 (primary tumor) and SW-620 (lymphnode metastasis) were derived from the same patient but belongs to different stages [25]. Thus we tested the two cell lines on apoptosis induction effect and the data indicated FPKc could induce SW-480 cells apoptosis more significantly than SW-620 cells. Taken together, we chose SW-480 cells as the subject to further determine the underlying mechanism in this paper. HEK-293 cells performed much lower injury effect than SW-480 cells (p<0.01), which was used to demonstrate the toxic effect of FPKc on normal cells. As for ES, we found its injury index was 35% in the dose of 24 µg/ml (10% of 240 µg/ml FPKc) after 48 h incubation. However, with 240 µg/ml FPKc treatment, SW-480 cells performed 65.20±2.34% viability loss in the same condition. Concerning all the above, we suppose FPKc might selectively damage some human colon cancer cells while with less effect on nonmalignant normal cells, and ES may play a significant role when FPKc exerted its antitumor function. Of course, we can’t exclude other active components that worked in this study.

Here we evaluated the anticancer activity of FPKc on SW-480 cells from two aspects: migration and growth inhibition. In cancer treatment, metastasis is one of the major challenges [26]. For CRC, the overall 5-year survival rate for patients with metastatic CRC is less than 10% [27]. Thus, preventing CRC metastasis is a key target to improve a patient's prognosis. In our study, FPKc has been proved to have an obvious anti-metastasis effect on SW-480 cells. To further evaluate the mechanism of the anti-metastasis effect by FPKc, we tested the expression of MMP-9 and MMP-2. It has been reported MMPs are vertical in tumor invasion and metastasis, because the formation of metastasis requires degradation of ECM [28]. It has been proved MMP-9 could facilitate tumor progression, invasion, metastasis angiogenesis [29]. The activation of MMP-9 is principally via MMP-2 and indirectly through an activation axis made up of TIMP-2 and MT1-MMP [30]. In this study, FPKc could distinctly inhibit the migration of SW-480 cells through down regulating the expression of MMP-2 and MMP-9 in SW-480 cells.

**Figure 13:**
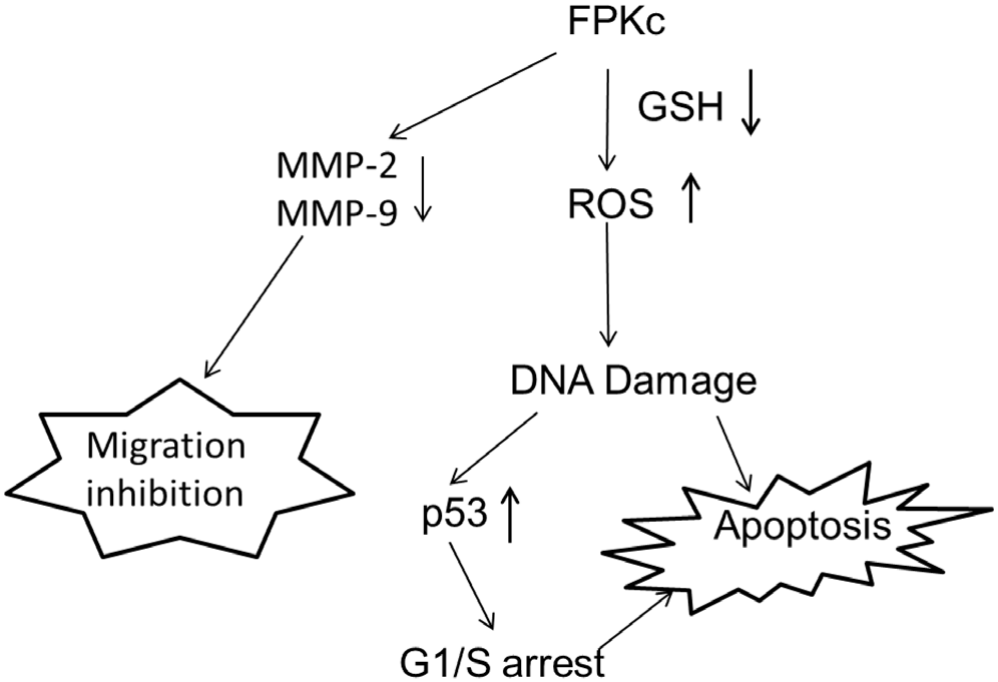
Proposed possible signal pathways for FPKc-induced apoptosis and migration inhibition in human colon cancer SW-480 cells.

It is commonly known that preventing tumorigenesis often involves signal transduction pathway modulation, resulting in cell cycle arrest and, eventually, apoptosis [19], [31]. To estimate the effect of FPKc treatment on the distribution of cells in the cell cycle, we performed DNA cell cycle analysis by flow cytometry. Our results suggested that FPKc and ES blocked proliferation of SW-480 cells by arresting the cells in G1 phase of the cell cycle. It is also widely recognized DNA damage could provoke the increase of P53 level to induce arrest within the G1 and G2 phase of the cell cycle, apoptosis, and DNA repair [32], [33]. Thus, in our study, we performed the DNA damage and P53 expression level. To our expect, after FPKc and ES treatment for 12 h, SW-480 cells performed prominent DNA fragmentation. And P53 was upregulated with FPKc and ES treating for 24 and 48 h. Therefore, we suggested that the growth inhibition of FPKc was associated with the G1 phase arrest, which was related to p53-dependent regulation in SW-480 cells (Figure 13).

Apoptosis is a normal physiologic process, which plays a significant role in homeostasis and development of the tissue in organism [34], and causing cell apoptosis in tumor tissue is the best stage for cancer therapy [35]. As we know, there are kinds of natural products having the ability to induce apoptosis in various human tumor cells [36]. Cells undergoing apoptosis always show the specific morphological changes, such as plasma membrane blebbing, chromatin condensation and apoptotic bodies formation [37]. In our study, HO staining revealed that cells treated with FPKc and ES for 48 h performed the distinct chromatin condensation in a dose-dependent manner. The percentage of the apoptotic cells was measured by Annexin V-FITC/PI staining. And our results showed after FPKc and ES treatment for 24 h, the proportion of apoptotic cells increased obviously. Moreover, caspases which are a family of cysteine proteases play a central role during the process of apoptosis [38]. Caspase-3, as one of the key executioners of apoptosis, is responsible for the proteolytic cleavage of many key proteins, such as the nuclear enzyme poly (ADP-ribose) polymerase (PARP), which are cleaved in many different systems during apoptosis [39]. Herein, our results showed cleaved-caspase 3 and cleaved-PARP were upregulated as the incubation time of FPKc and ES increased from 12 to 48 h. P53, as a tumor suppressor, could also induce apoptosis through targeting Bcl-2 family: up-regulating pro-apoptotic protein Bax and down-regulating anti-apoptotic protein Bcl-2 [40]. In the present study, our data showed that a decrease in Bcl-2 expression occurred accompanied with P53 expression increased in SW-480 cells which were treated with FPKc and ES. Thus we could conclude that FPKc induced apoptosis might belong to caspase dependent manner and P53 might also play an important role in this pro-apoptosis process (Figure 13).

Previous studies indicate that the production of ROS is vertical in the pro-apoptosis effect of traditional Chinese medicine [23]. Thus ROS generation was performed in this study. The results revealed that after incubation with FPKc and ES for 3 h and 6 h, the accumulation of cellular ROS was increased extremely, suggesting that ROS might be of great significance in FPKc induced apoptosis (Figure 13). Cellular GSH, as the principal detoxifying system, is capable of scavenging ROS and maintaining the redox state of cellular thiols [41]. Depletion of cellular thiols may potentially lead to oxidative stress which means over-production of ROS can be secondary to intracellular GSH depletion [42], [43]. What’s more, GSH may modulate the transcription of specific genes, regulate redox-sensitive signal transduction and cell proliferation, apoptosis [44]. Thus in our study, the concentration of intracellular GSH after FPKc and ES treating on SW-480 cells was performed. And the results showed GSH level was much lower than control after FPKc and ES treatment for 3 h and 5 h, which inferred FPKc induced the ROS accumulation through decreasing intracellular GSH content.

Moreover, to further confirm the finding that the apoptotic effect of FPKc was mediated by ROS, antioxidants NAC was also employed. The results revealed NAC could decrease intracellular ROS generation, reverse DNA damage, relieved cell viability loss and apoptosis caused by FPKc treatment. These results indicated that ROS was involved in FPKc-induced apoptosis in SW-480 cells (Figure 13).

## Conclusion

Taken together, our data showed that FPKc could inhibit cell migration, induce ROS-dependent apoptosis and cause P53 mediated G1 phase arrest in human colorectal cancer SW-480 cells. And, ES as one of the main components of FPKc might be involved in these processes. The obtained findings provide rational insight for further evaluation of FPKc as a safe, efficient and selectively agent for treating and preventing human colon cancer. To clarify the specific signal pathway, we still have long way to go.

## References

[1] Jemal A, Bray F (2011) Center MM, Ward E, Forman D, et al (2011) Global cancer statistics. CA Cancer J Clin 61(2): 69–90. 21296855 10.3322/caac.20107

[2] Siegel R, Naishadham D, Jemal A (2013) Cancer Statistics, 2013. CA Cancer J Clin 0: 000–000. 10.3322/caac.21166 23335087

[3] Lu JB, Sun XB, Dai DX, Zhu SK, Duan WJ, et al (2003) Epidemiology of gastroenterologic cancer in Henan Province, China. World J Gastroenterol 9: 2400–2403. 14606064 10.3748/wjg.v9.i11.2400 PMC4656509

[4] Cragg GM, Newman DJ (2005) Plants as a source of anti-cancer agents. J Ethnopharmacol 100: 72–79. 16009521 10.1016/j.jep.2005.05.011

[5] Ying JZ, Mao XL, Ma QM, Zong YC, Wen HA (1987) Illustrated handbook for medicinal fungi from China. Beijing: Science Press. 120, 128,172, 218p.

[6] Hogberg N, Holdenrieder O, Stenlid J (1999) Population structure of the wood decay fungus Fomitopsis pinicola. Heredity 83: 354–360. 10504434 10.1038/sj.hdy.6885970

[7] Usui T, Shinkai K, Satoh H, Iwasaki Y, Mizuno T (1982) Studies on Host mediated Antitumor Polysaccharides. Part V. Chemical structure and antitumor activity of a water-soluble Glucan isolated from Tsugasarunokoshikake, the fruit body of Fomitopsis pinicola. Shizuoka Daigaku Nogakubu Kenkyu Hokoku 32: 29–40.

[8] Yoshikawa K, Inoue M, Matsumoto Y, Sakakibara C, Miyataka H, et al. (2005) Lanostane triterpenoids and triterpene glycosides from the fruit body of Fomitopsis pinicola and their inhibitory activity against COX-1 and COX-2. Journal of Natural Products 68(1): 69–73, 29–40. 10.1021/np040130b 15679320

[9] Keller AC, Maillard MP, Hostettmann K (1996) Antimicrobial steroids from the fungus Fomitopsis pinicola. Phytochemistry 414: 1041–1046. 10.1016/0031-9422(95)00762-8 8728714

[10] Guler P, Akata I, Kutluer F (2009) Antifungal activities of Fomitopsis pinicola (Sw: Fr) Karst and Lactarius vellereus(Pers.) Fr. African Journal of Biotechnology 8(16): 3811–3813.

[11] Ren G, Liu XY, Zhu HK, Yang SZ, Fu CX (2006) Evaluation of cytotoxic activities of some medicinal polypore fungi from China. Fitoterapia 77(5): 408–410. 16797143 10.1016/j.fitote.2006.05.004

[12] Wu HT, Lu FH (2014) In Vivo and In Vitro Anti-Tumor Effects of Fungal Extracts. Molecules 19: 2546–2556 24566320 10.3390/molecules19022546 PMC6270758

[13] Rosecke J, Konig WA (1999) Steroids from the fungus Fomitopsis pinicola. Phytochemistry 52(8): 1621–1627.

[14] Rosecke J, Konig WA (2000) Constituents of various wood-rotting basidiomycetes. Phytochemistry 54(6): 603–610. 10963454 10.1016/s0031-9422(00)00165-5

[15] Azahata Y, Sugiyama K (1994) Anti-tumor promoting effect of Kampo formulations on rat urinary bladder carcinogenesis(VI). Effect of ergosterol on rat urinary bladder carcinogenesis in long-term assay. Wakan Iyakugaku Zasshi 11: 344–345.

[16] Azahata Y, Yokota MK (2000) Anti-tumor promoting effect of an active component of polyporus, ergosterol and related compounds on rat urinary bladder carcinogenesis in a short-term test with concanavalin. A. Biol. Pharm. Bull 23: 1298–1302. 10.1248/bpb.23.1298 11085355

[17] Cheng X, Xiao Y, Wang X, Wang P, Liu Q, et al (2012) Anti-tumor and pro-apoptotic activity of ethanolic extract and its various fractions from Polytrichum commune L.ex Hedw in L1210 cells. J Ethnopharmacol 143: 49–56. 22687253 10.1016/j.jep.2012.05.054

[18] Krysko DV, Berghe TV, D’Herde K (2008) Apoptosis and necrosis: detection, discrimination and phagocytosis. Methods 44: 205–221. 18314051 10.1016/j.ymeth.2007.12.001

[19] Leventis PA, Grinstein S (2010) The distribution and function of phosphatidylserine in cellular membranes. Annu Rev Biophys 39: 407–427. 20192774 10.1146/annurev.biophys.093008.131234

[20] Shi X, Zhao Y, Jiao Y, Shi T, Yang X (2013) ROS-dependent mitochondria molecular mechanisms underlying antitumor activity of pleurotus abalonus acidic polysaccharides in human breast cancer MCF-7 cells. PLoS ONE 8(5): e64266. 23691187 10.1371/journal.pone.0064266 PMC3653930

[21] Li L, Lu N, Dai Q, Li Z, Dai Y, et al (2011) Q. GL-V9, a newly synthetic flavonoid derivative, induces mitochondrial-mediated apoptosis and G2/M cell cycle arrest in human hepatocellular carcinoma HepG2 cells. Eur. J. Pharmacol. 670: 13–21. 10.1016/j.ejphar.2011.08.054 21944925

[22] Zhang H, Zhang M, Yu L, Zhao Y, Yang XB, et al (2012) Antitumor activities of quercetin and quercetin-5, 8-disulfonate in human colon and breast cancer cell lines. Food Chem. Toxicol 50: 1589–1599. 10.1016/j.fct.2012.01.025 22310237

[23] Li-Weber M (2013) Targeting apoptosis pathways in cancer by Chinese medicine. Cancer Letters 332: 304–312. 20685036 10.1016/j.canlet.2010.07.015

[24] Townsend DM, Tew KD, Tapiero H (2003) The importance of glutathione in human disease. Biomed. Pharmacother 57: 145–155. 10.1016/s0753-3322(03)00043-x PMC6522248 12818476

[25] Leibivitz A, Stinson JC, McCombs WB III, McCoy CE, Mabry ND, et al (1976) Classification of human colorectal adenocarcinoma cell lines. Cancer Res. 36: 4562–4569. 1000501

[26] Lu KW, Chen JC, Lai TY, Yang JS, Chung JG, et al (2011) Gypenosides inhibits migration and invasion of human oral cancer SAS cells through the inhibition of matrix metalloproteinase-2-9 and urokinase-plasminogen by ERK1/2 and NF-kappa B signaling pathways. Hum Exp Toxicol 30: 406–415. 20511288 10.1177/0960327110372405

[27] Goldberg RM (2005) Advances in the Treatment of Metastatic Colorectal Cancer. The Oncologist 10: 40–48. 16368870 10.1634/theoncologist.10-90003-40

[28] Rowe RG, Weiss SJ (2009) Navigating ECM barriers at the invaive front: the cancer cell-stroma interface. Annu Rev Cell Dev Biol 25: 567–595. 19575644 10.1146/annurev.cellbio.24.110707.175315

[29] Abba M, Patil N, Allgayer H (2014) MicroRNAs in the Regulation of MMPs and Metastasis. Cancers. 6: 625–645. 10.3390/cancers6020625 PMC4074795 24670365

[30] Groblewska M, Siewko M, Mroczko B, Szmitkowski M (2012) The role of matrix metalloproteinases (MMPs) and their inhibitors (TIMPs) in the development of esophageal cancer. Folia Histochem. Cytobiol. 50: 12–19. 10.2478/18691 22532131

[31] Hafeez BB, Siddiqui IA, Asim M, Malik A, Mukhtar H, et al (2008) A dietary anthocyanidin delphinidin induces apoptosis of human prostate cancer PC3 cells in vitro and in vivo: involvement of nuclear factor-κB signaling. Cancer Res 68: 8564–8572. 18922932 10.1158/0008-5472.CAN-08-2232 PMC3149885

[32] Lakin ND, Jackson SP (1999) Regulation of p53 in response to DNA damage. Oncogene 18: 7644–7655. 10618704 10.1038/sj.onc.1203015

[33] He N, Shi X, Zhao Y, Tian L, Wang D, et al (2013) Inhibitory effects and molecular mechanisms of selenium-containing tea polysaccharides on human breast cancer MCF-7 cells. J Agric Food Chem 61: 579–588. 23270479 10.1021/jf3036929

[34] Kerr JF, Winterford CM, Harnon BV (1994) Apoptosis–its significance in cancer and cancer therapy. Cancer 73: 2013–2026. 8156506 10.1002/1097-0142(19940415)73:8<2013::aid-cncr2820730802>3.0.co;2-j

[35] Thompson CB (1995) Apoptosis in the pathogenesis and treatment of disease. Science 267: 1456–1462. 7878464 10.1126/science.7878464

[36] Taraphdar AK, Roy M, Bhattacharya RK (2001) Natural products as inducers of apoptosis: Implication for cancer therapy and prevention. Curr. Sci. 80: 1387–1396.

[37] Villanueva A (2005) Morphological criteria to distinguish cell death induced by apoptotic and necrotic treatments. Apoptosis 10: 201–208. 15711936 10.1007/s10495-005-6075-6

[38] Thornberry NA, Lazebnik Y (1998) Caspases: enemies within. Science 281: 1312–1316. 9721091 10.1126/science.281.5381.1312

[39] Cohen GM (1997) Caspases: the executioners of apoptosis. Biochem. J. 326: 1–16. 10.1042/bj3260001 PMC1218630 9337844

[40] Polyak K, Yong X, Zweier JL, Kinzler KW, Vogelstein B (1997) A model for p53-induced apoptosis. Nature (London) 389: 300–305. 9305847 10.1038/38525

[41] Sies H (1999) Glutathione and its role in cellular functions. Free Radic Biol Med. 27: 916–921. 10.1016/s0891-5849(99)00177-x 10569624

[42] Meister A, Anderson ME (1983) Glutathione. Annu Rev Biochem 52: 711–760. 6137189 10.1146/annurev.bi.52.070183.003431

[43] Chang HH, Guo MK, Kasten FH, Chang MC, Huang GF, et al (2005) Stimulation of glutathione depletion, ROS production and cell cycle arrest of dental pulp cells and gingival epithelial cells by HEMA. Biomaterials 26: 745–753. 15350779 10.1016/j.biomaterials.2004.03.021

[44] Ratan RR, Murphy TH, Baraban JM (1994) Oxidative stress induces apoptosis in embryonic cortical neurons. J Neurochem. 62: 376–379. 10.1046/j.1471-4159.1994.62010376.x 7903353

